# The Relation the House Fly Bears to Typhoid Fever and Other Infectious Diseases

**Published:** 1910-08

**Authors:** J. W. Palmer

**Affiliations:** Ailey, Ga.


### THE RELATION THE HOUSE FLY BEARS TO TYPHOID FEVER AND OTHER INFECTIOUS DISEASES.

BY DR. J. W. PALMER, AILEY, GA.

I have written on this subject again to show the new developments, proofs, and progress made during the last twelve months relative to the dangers of the house fly, and also, in order that the general interest may be increased, the subject continually agitated and kept vividly before our minds. The history of the Association has been that these important subjects die out as soon as they cease to be interesting before the meetings. For instance, the hook worm subject was getting to be non-interesting to the meetings until the million dollar gift gave it new life.

Scientists of all countries have agreed on one thing, namely, that flies are carriers of disease germs.

*Typhoid Fever.*—Physicians, scientists, and the laity in all countries have agreed that the house fly carries typhoid bacilli to our food and drink. They differ only as to the extent and this is because we have no scientific means of definitely estimating the number of cases infected by them.

In my paper last year, I stated that the house fly was responsible for 85% of the typhoid fever cases in the cities and 95% in the rural districts. Some thought this a very wild and radical statements, but during the last twelve months the best authorities

Read before the Ga. Med. Ass'n. at Athens, Ga. 9pril 20-23, 1910.

claim the flies have been proven to be responsible for 30% more cases than thought last year. It is only a question of a few years until the researches, investigations, and experiments along this line will convince the entire world that the eradication of the house fly will eliminate typhoid fever.

Each year in this country typhoid fever claims fifty thousand victims out of the quarter of a million of subjects who are stricken with it annually and costs the country $50,000,000. It causes in one year more deaths than yellow fever in fifty years, however, the government protects us from yellow fever and leaves us unprotected from typhoid fever. There are more typhoid fever and house flies in the United States than in any other civilized country. Hence, Surgeon-General Wyman has named it the "National Disgrace."

The chronic typhoid bacillus carrier is the source of so many cases of unknown infection carried by the fly, and it is these that make the flies so dangerous. These individuals are those who once had typhoid fever and continually and forever liberate these germs equal to the time when they were stricken with fever. These carriers have been proven without a doubt to the satisfaction of everybody. It is claimed that five out of a hundred who have typhoid fever become chronic typhoid bacillus carriers.

From observations, I certainly know that the house flies are practically responsible for the rural typhoid fever and I believe that experiments, close study of the subject, and keen observations will in a short time show that, if not directly, they are indirectly responsible for most of the city typhoid.

Most all cities have their sewage emptying into some body of water. Some cities claim, because their sewage empties into tidal water, it is innocious, on the grounds that sewage discharges emptying into tidal waters are rendered innocious by the natural actions of moving tidal water.

This has all been proven to be erroneous because the floating excreta and the flies caught on it have been examined and the typhoid and dysentery bacilli found on them; besides typhoid fever and summer bowel diseases are so much more frequent in this section of these cities and especially when the number of flies is at the greatest. This floating excreta is sought by the flies and after feasting on it they go to the nearest restaurants, homes and hotels to feast on food, milk and water and infect the same with deadly germs, thereby producing numberless cases of typhoid fever which are attributed to the water. In the cities where the sewage discharges empty into rivers and other streams the swift current forces the suspended matter to the edges of the streams to lodge against the banks and eddy there, which furnishes infection and breeding places for flies. And from these places the flies go to the various parts of the city to scatter the germs with which they are contaminated. The city says, "Poor sewage," overlooking the fact that the fly is the cause.

In the cities you will find very few who keep the closets fly-proof. You find the sinks, the underground or imperfectly drained cess pools, the back yards of homes, all good places to breed flies and to receive infection. The chronic typhoid bacillus carrier comes along and disseminates the typhoid bacilli in the non-fly-proof closets swarming with flies. From these closets the flies go out all over the city, not only infecting the food and drink at places of eating, but infecting other closets, cess pools, sinks and back yards where other flies become contaminated and go out over the city infecting more food and drink, and so it goes ad infinitum, and we have a big city epidemic of typhoid fever due to flies, and the city bacteriologist calls it impure water or milk.

The courts have passed upon the question of damages for a sufferer from typhoid fever who could trace his illness to flies feeding upon the filth of sewage. Sometime ago a man living in Germantown, Philadelphia, recovered heavy damages from the city for his illness which he proved was caused by a stream flowing through his yard which had been polluted by sewage from a house tenanted by a typhoid patient. The defense relied upon the proof that the plaintiff had neither drunk nor bathed in the stream, but an entomologist convinced the jury that he had contracted the disease through the medium of flies which had carried the infection from the stream to the food exposed to their visits in his house. About the only way I can see that the flies are not responsible for city typhoid is when the infection is due to leak in pipes and direct infection from contact. All the infected dairies that cause typhoid epidemics are infected through the flies.

*Intestinal Summer Bowel Troubles.*—The house fly is now considered the principal cause of diarrhœal and intestinal summer bowel trouble. The deaths from these diseases increase and decrease with the general prevalence of flies. For instance, in New York from November to May there are no flies and no diarrhœal diseases, but as soon as the flies appear these diseases appear and both increase at the same ratio, and in August when flies are most numerous these diseases are at their height, after which they decrease with the falling off of the flies. Seven thousand children alone die annually in New York City from diarrhœal diseases. Baltimore, Philadelphia, and Chicago have equally as great per cent., and in the rural districts the same is true as in the city except, I believe, the per cent. is greater. The fall and rise in flies and these diseases of course correspond also to the temperature curve. It has been the custom for us to attribute this to high temperature, while it has nothing to do with it, except lower the vitality, while the true cause is due to infection from germs carried by flies. The temperature might lower the vitality ever so much, but if they did not come in contact with the germs they could not have the disease, because extreme cold lowers the vitality as much as extreme heat, but as there are no flies in the winter to carry the germs, we scarcely have any diarrhœal troubles in the winter.

The reason so many are slow to accept the fly theory is that they have failed to devote any time to the subject and give it any serious thought. About one month ago I wrote a letter to practically all the State Boards of Health and some boards in the large cities, asking that they give me their opinion as to the extent the house fly is responsible for typhoid fever. I received a reply from all, I think, and they claim the fly to be a great factor in the dissemination of typhoid bacilli; some believed they were the principal cause and others thought other causes more important. There was one particularly noticeable and interesting feature in these replies: those who said they had not given the subject much thought, nor made any experiments or investigations, were those who thought the flies were not the principal cause, while those who had given the subject thought, with experiments and investigation, claimed that the flies were the principle cause.

Doctor J. N. Hurty, Secretary Indiana State Board of Health, states that with only a few investigations he believes that the greater portion of typhoid fever has been carried by flies. Doctor S. J. Crumbine, Secretary Kansas State Board of Health, says he has traced typhoid epidemics to the fly so often where water, milk, and other things were not the cause, that it is his positive conviction that the flies are responsible, at the very least, for 50% of the cases in his State. Dr. W. C. Hassler, San Francisco, says he installed 6,000 concrete horse-stable floors and that in so doing destroyed practically the entire breeding places of flies and, as a result, he finds a great reduction in all communicable diseases and that typhoid fever and summer bowel trouble is practically a thing of the past. He further states that typhoid fever had been the disease principally considered by the Board of Health and showed by experiments in his own laboratory that nearly every case of infection was fly born.

The Board of Health in Chicago claims the fly is responsible for the largest per cent. of typhoid fever cases and has been giving it a close study since Doctor Alice Hamilton, of Chicago, showed in 1902 that one of their largest epidemics was due to the spread of the bacilli by flies.

There was an epidemic of parotyphoid fever at Weyers Cave, Va., last summer. The epidemiological studies showed that it was not water, milk, shell fish, fresh vegetables, fruits or other foods that carried the infection, therefore it must have been flies.

Doctor G. M. Kober was one of the first to call attention to the danger of house flies in 1895 and he again called attention to it when he read his paper on "Conservation of National Resources" before the Governors' Convention held at the White House in 1908. This Association has the honor of having as one of its members a physician who was the first to show conclusively that the flies were responsible for typhoid fever. This is Doctor R. P. Izlor, of Waycross, who wrote a paper showing that the typhoid epidemic in Tampa, Fla., was due to the flies. This paper was read as part of his presidential address in Jacksonville, Fla., in 1898.

During the past season, I treated typhoid fever in several families and I took particular notice that the families which controlled the flies as directed had no new cases to develop, while the families which did not control the flies had any where from one to four cases to develop in each family.

The reason artificially fed babies are more susceptible to bowel trouble is due to the fact that flies swarm on the nipple of their nursing bottle while mothers keep flies from their breasts.

Flies are also responsible for a certain amount of the following diseases: Tuberculosis, diphtheria, scarlet fever, small-pox, pneumonia, tetanus, plague, Asiatic cholera, felariosis, gonorrhea, and certain skin, wound, and eye infections.

Gentlemen, we can not be too severe in the arraignment of the house fly, because he has cost mankind more health, strength and happiness than war. War is a terrible thing, awful beyond description, and yet flies have brought more misery than war, yet our government spends millions on war and not one cent on flies. As carriers of contagion their responsibility is unlimited; we are just beginning to realize their danger as disseminators of disease germs. Many an obscure case of infection might be placed to their credit and many a break in the family circle might give silent testimony to the part the flies have played in the drama. When we wonder how baby got bowel trouble; how Johnnie got typhoid; how Jennie got consumption; how Fannie got blood poison; or how Grandma got dysentery, it is all solved by the disseminating flies. The flies are regarded as more dangerous than the tiger or cobra. Worse than that he is, at least in our climate, much more to be feared than the mosquito and may easily be classed the world over as the most dangerous creature on earth.

Since my last paper on the fly there has been a general increase in interest in the subject and an awakening of the public, not only all over the United States, but in England and in certain English colonies as well. In Glasgow and Liverpool they are carrying on a crusade against the flies. The English government is awakening to this fact and in their parliamentary vote of the present year on disease investigation the house fly was included. The Liverpool of tropical medicine has been continued, and the work of the Merchants Association in New York, as you doubtless know, was carried through the summer of 1909. One of the most encouraging developments of recent date has been the adoption of the house fly as a subject for campaign by the American Civic Association with their headquarters at Washington, D. C. Mr. Edward Hatch, Jr., is now with them, saying that the Merchants Association was confined to New York State and that the widespread popularity of the anti-fly movement made the organization of a national committee necessary and therefore he went with the American Civic Association. This new committee will endeavor to enlist the co-operation of every State in the Union and to carry the campaign all over the world. This committee has secured moving pictures illustrating the development of the house fly and its agency in the carriage of disease. These moving pictures will be placed in the hands of every exhibitor of motion pictures, especially five-cent shows, of which there are more than 7,000 in the United States. This committee is in co-operation with other forces, and each State wants to place a special man in each State to go over it with these shows lecturing and demonstrating the dangers of the house flies, and especially should this be done in the schools of the State.

There are 3,000 Women's Clubs in the United States with membership of 150,000 who are rendering the anti-fly movement their enthusiastic and earnest co-operation. Doctor L. C. Howard, Chief Entomologist of the Bureau of Agriculture, Washington, D. C., writes me that his correspondence within the last two weeks has indicated a tremendous widespread interest all over the country in the general subject. Letters are received every day from people who wish to organize campaigns in their respective communities.

